# Clinical Outcomes and Correction Rates of Valgus and Varus Deformities Treated with Temporary Hemiepiphysiodesis Using Tension Plates: A Retrospective Cohort Study

**DOI:** 10.3390/medicina62010165

**Published:** 2026-01-14

**Authors:** Manuel Gahleitner, Tobias Gotterbarm, Lorenz Pisecky

**Affiliations:** Department for Orthopedics and Traumatology, Kepler University Hospital GmbH, Johannes Kepler University Linz, 4040 Linz, Austria

**Keywords:** genu valgum, genu varum, tension band plate, growth modulation, hemiepiphysiodesis, knee, pediatric orthopedics

## Abstract

*Background and Objectives*: Coronal plane deformities of the knee, particularly genu valgum and varum, represent common indications for guided growth in pediatric orthopedics. This study evaluates the clinical and radiographic outcomes of temporary hemiepiphysiodesis using tension-band plates in skeletally immature patients and identifies factors associated with successful correction. *Materials and Methods*: A retrospective review was conducted on patients treated with tension-band plate hemiepiphysiodesis for knee valgus or varus deformities between 2012 and 2023. Inclusion required open physes, pre- and postoperative full-length radiographs, and follow-up until implant removal or skeletal maturity. Mechanical axis parameters (mLDFA, mMPTA) were compared pre- and postoperatively, and correction rates were calculated. Idiopathic cases were analyzed separately from those with neurological or osteological disorders. *Results*: Sixty-six limbs were included (51 valgus, 15 varus). In the idiopathic subgroup, significant correction was achieved, with mLDFA improving by +5.19° and mMPTA by −1.88°, corresponding to annual correction rates of 4.75°/year and −1.74°/year, respectively (*p* < 0.001). Regression analysis showed no significant predictive value of age or treatment duration for total correction. Patients with pathological physes demonstrated inconsistent outcomes, often requiring additional procedures. No major complications occurred. *Conclusions*: Temporary hemiepiphysiodesis using tension-band plates is a safe, minimally invasive, and highly effective method for correcting idiopathic valgus deformities in growing children, with correction rates comparable to the existing literature. Outcomes in patients with neurological or osteological comorbidities remain less predictable, underscoring the need for individualized planning and close follow-up.

## 1. Introduction

Coronal deformities of the lower extremities, particularly genu valgum and varum, are frequently encountered in pediatric orthopedic practice. While physiologic valgus alignment is a normal part of skeletal development and typically resolves by the age of 7–8 years, persistent or pathologic valgus deformities beyond this age can lead to significant mechanical axis deviation [1]. This may result in uneven load distribution across the knee joint, abnormal gait mechanics, progressive joint degeneration, and, eventually, premature osteoarthritis if left uncorrected. In cases where the deformity is severe, progressive, or symptomatic, surgical intervention may be necessary.

Manipulation of growing bone by an operative or non-operative method is an old concept and widely used in pediatric orthopedic practice, for example, in Pavlik harness treatment for developmental dysplasia of the hip or using Cheneau-brace in the case of scoliosis [2].

Temporary hemiepiphysiodesis or epiphyseodesis has emerged as a well-established technique for correcting coronal plane deformities or leg-length-discrepancy in skeletally immature patients. There is ample evidence in the literature to suggest that bone remodeling and growth can be modulated by applying mechanical forces [3]. Among the various methods developed for guided growth, the use of a tension-band plate—commonly known as the Eight-Plate^®^—has gained widespread acceptance due to its simplicity, minimal invasiveness, and reversibility. Introduced by Stevens in 2007, the Eight-Plate^®^ (Orthofix, Lewisville, TX, USA) device applies asymmetric growth inhibition across the physis, enabling gradual correction of angular deformities during the remaining growth period without causing permanent damage to the growth plate [4]. Despite the growing popularity of tension-plate hemiepiphysiodesis, there remains considerable variability in reported correction rates, treatment durations, and clinical outcomes. Several factors may influence the efficacy of this method, including the patient’s age at surgery, the severity and location of the deformity, the rate of skeletal growth, and the timing of implant removal [5,6]. While earlier intervention allows a more predictable and complete correction due to higher remaining growth potential, it may also increase the risk of rebound growth or overcorrection, particularly in younger patients [7]. Choi et al. showed that the rebound group had a younger age and faster correction rate than those in the non-rebound group. In addition, the correction rate for deformity was a significant risk factor for the rebound phenomenon after hemiepiphysiodesis using the tension band plate. Close monitoring after implant removal is required for children who have a rapid correction rate over 7°/year [8].

Conversely, delayed treatment may reduce the window for effective correction and lead to residual deformity at skeletal maturity [9]. Given the importance of optimizing the timing and duration of treatment, further clinical evidence is needed to guide decision-making in the use of tension-plate hemiepiphysiodesis. To date, only limited studies have systematically evaluated the relationships between patient age, initial deformity magnitude, duration of treatment, and achieved correction in a uniform patient population undergoing valgus correction. Agarwal et al. found that there are influences from several factors. Valgus and femoral deformities correct at a faster rate in younger children. On multivariate analysis, the location and the duration of treatment showed significant associations with the correction rate [10].

Temporary hemiepiphysiodesis is also commonly applied to correct coronal plane deformities associated with metabolic or osteological disorders. Although widely adopted in clinical practice, published results are variable, and current evidence does not allow for uniform conclusions regarding predictability and effectiveness across these heterogeneous conditions [11,12,13].

The present retrospective study aims to assess the clinical and radiographic outcomes of temporary hemiepiphysiodesis using tension-plates in a cohort of skeletally immature patients with genu valgum or genu varum. Specifically, we examine the degree of angular correction achieved, the duration of treatment required, and the impact of patient-related factors such as age and initial deformity angle on treatment outcomes. By identifying key predictors of successful correction, this study seeks to contribute to the optimization of treatment planning and timing in the management of lower limb angular deformities using guided growth techniques.

## 2. Materials and Methods

We performed a retrospective review of prospectively collected records of all patients undergoing guided growth surgery for coronal plane angular deformity of the knee using the tension band plate at our urban tertiary national referral center from 2012 to 2023. Ethical approval was obtained from the local institutional review board (EK1224/2023 Version 5).

To qualify for enrollment in this study, patients were required to present with a coronal plane angular deformity of the knee that was managed using tension band plating. Each participant needed to have at least one preoperative full-length, weight-bearing anteroposterior radiograph of the affected limb, along with postoperative imaging. The interval between imaging studies was determined by the treating surgeon, usually 6 months. Two different two-hole plate systems were utilized: the Eight-Plate^®^ (Orthofix, Lewisville, TX, USA) and the Pediplate^®^ (Orthopediatrics, Warsaw, IN, USA). Over time, the Pediplate^®^ became the preferred option and was used more consistently.

Inclusion criteria consisted of:

1. Children with open physis, confirmed radiographically.
2. Diagnosis of coronal plane angular deformity based on assessment of the lower limb mechanical axis on standing anteroposterior radiographs, regardless of underlying cause.
3. Undergoing hemiepiphysiodesis of the distal femur, proximal tibia, or both.
4. Availability of follow-up until skeletal maturity or until implant removal.
5. Complete medical records and clinical data accessible for review.

When a patient received guided growth surgery that was monitored until skeletal maturity, but later experienced a recurrence of deformity requiring another guided growth procedure, these were considered two distinct interventions. The initial surgery was regarded as successful, with the subsequent deformity classified as a recurrence or “rebound.” Rebound was defined as a recurrent angular deformity of ≥5° in the previously corrected segment following implant removal during further growth [8].

Similarly, if both limbs were corrected for deformity, each side was counted as a separate treatment. When guided growth was performed simultaneously on the tibia and femur, we counted this as two physes for statistical results.

### 2.1. Clinical Examination

During supine examination, the intermalleolar distance exceeded three fingerbreadths of the examiner in case of valgus deformity, prompting acquisition of a standing full-length radiograph for lower limb alignment assessment. In case of varus deformity, an intercondylar distance of three fingerbreadths was also used as indication to do a radiograph.

### 2.2. Radiographic Assessment

**Figure 1:**
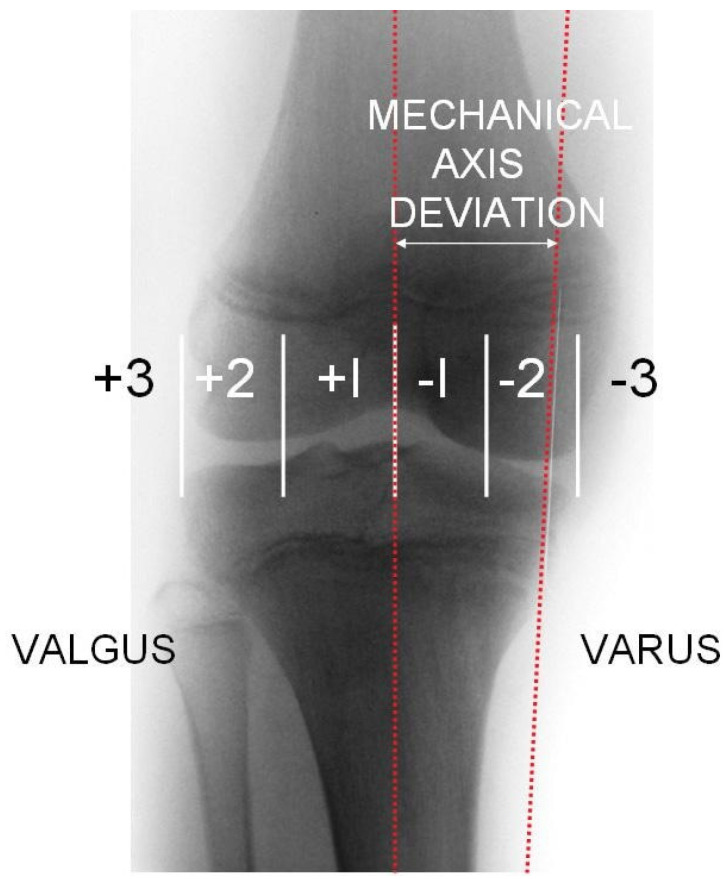
Classification by Muller and Muller-Faber [14].

**Figure 2:**
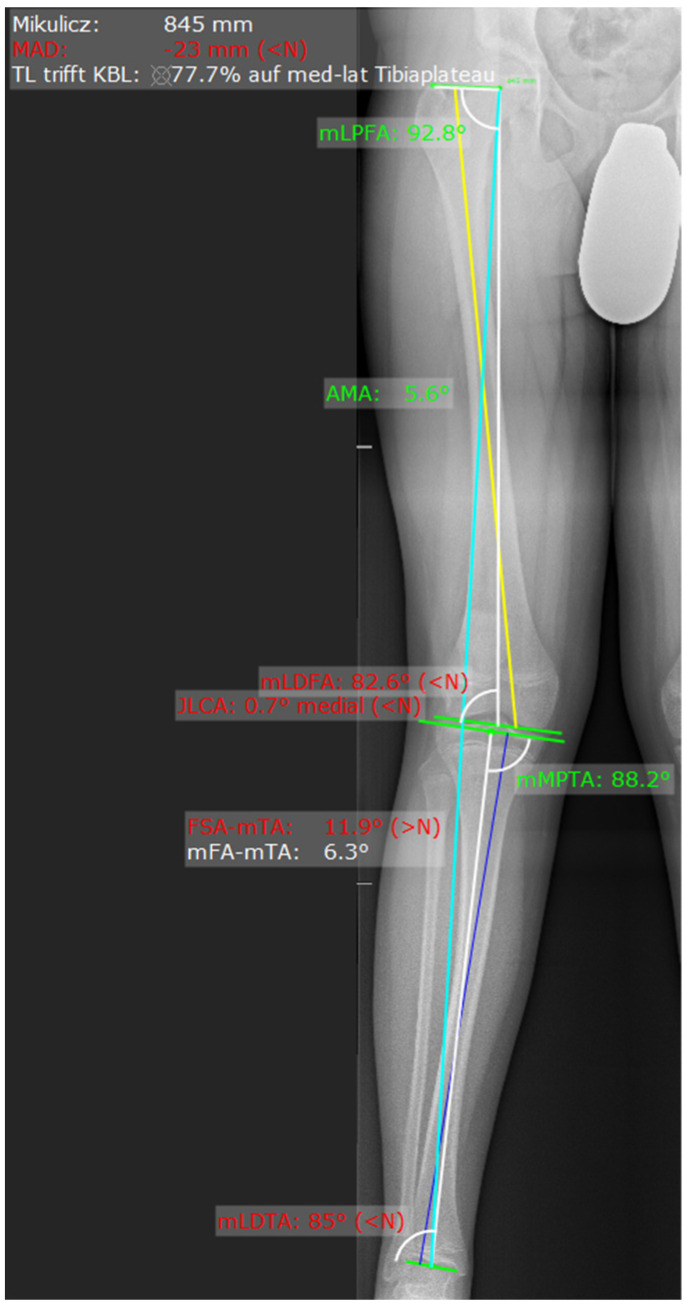
Measurement via MediCAT. Blue line from the femoral head to the talus represents the Mikulicz line. The yellow line represents the axis of the femur and the dark blue line the axis of the tibia.

On the radiograph, the mechanical axis (Mikulicz line) was drawn from the center of the femoral head to the center of the talus, and the lateral and medial compartment of the knee joint was divided into three equal zones. If the mechanical axis intersected the middle third, a relative surgical indication was given; if it intersected the lateral or medial third, an absolute surgical indication was established (Figure 1). This technique and classification was described by Muller and Muller-Faber [14]. To determine whether the distal medial femur or the proximal medial tibia should be addressed, a deformity analysis was performed by measuring the mechanical lateral distal femoral angle (mLDFA) and the mechanical medial proximal tibial angle (mMPTA) (Figure 2). The location of the deformity was identified based on these measurements (Table 1). Radiographic measurements were performed independently by the attending orthopedic surgeon and a board-certified radiologist. The indication for surgery was based on the measurements obtained by the surgeon. For the purpose of this study, the reported values represent the mean of the measurements obtained by the surgeon and the radiologist.

### 2.3. Surgical Timing Determination

**Figure 3:**
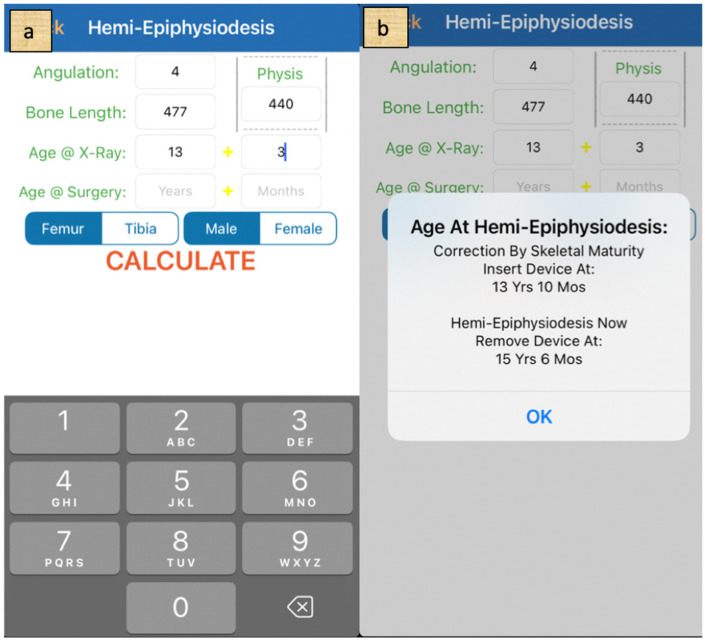
Calculation via mobile application. (**a**)—All the necessary data are given. (**b**)—The result defines the best time for surgery.

Following radiographic evaluation, the optimal timing for surgery was calculated using the multiplier method in conjunction with the Paley growth prediction system, with calculations performed via a dedicated mobile application for Apple^®^ (Figure 3a,b). For logistical reasons, surgery was scheduled several months earlier than the calculated optimal date to mitigate the risk of delay or suboptimal outcomes in the event of unforeseen circumstances, such as patient illness.

### 2.4. Surgical Technique

**Figure 4:**
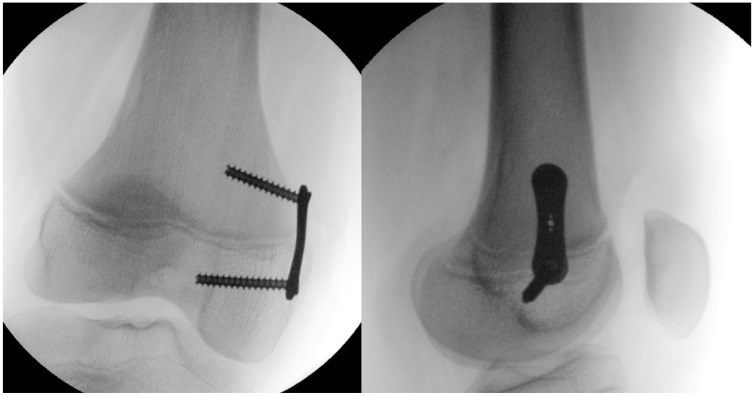
Postoperative biplanar radiographic evaluation of a temporary medial distal femoral hemiepiphysiodesis.

All procedures were performed with the patient in the supine position under fluoroscopic guidance. First, the physis was localized and marked with a Kirschner wire, with radiographic confirmation in two planes. The periosteum was then exposed to prepare the plate bed, after which the tension band plates was advanced over the positioned Kirschner wire and secured using cancellous screws (Figure 4).

### 2.5. Postoperative Protocol

Postoperatively, routine radiographic and clinical follow-up examinations were performed at six-month intervals. Upon achieving the desired correction and with remaining growth potential, the tension band plates were removed in a timely manner. If skeletal growth was complete, patients were given the option to decide independently on implant removal.

For subgroup assignment, neurological or osteological disorders were defined as pre-existing conditions with a documented diagnosis known to affect neuromuscular function, bone quality, or skeletal growth. This included neurological diseases, metabolic bone disorders, skeletal dysplasias, and deformities secondary to trauma.

### 2.6. Statistics

All statistical analyses were performed using IBM SPSS Statistics (version 26; IBM Corp., Armonk, NY, USA). Continuous variables were tested for normality using the Shapiro–Wilk test. Normally distributed data are presented as mean ± standard deviation (SD); non-normal data are presented as median with interquartile range (IQR). Pre- and postoperative measurements of the mechanical lateral distal femoral angle (mLDFA) and mechanical proximal tibial angle (mMPTA) were compared using paired *t*-tests for parametric data and Wilcoxon signed-rank tests for non-parametric data. Effect sizes were expressed as paired Cohen’s d. Annual correction rates (°/year) were calculated by dividing the absolute angular change by the treatment duration in years. As some patients contributed more than one physis or limb, a robustness analysis was performed in the idiopathic subgroup to account for potential within-patient clustering. Pre–post changes in the relevant mechanical angle (Δ = postoperative − preoperative) were analyzed using a linear mixed-effects model with a random intercept for patient.

To evaluate potential predictors of angular correction, segment-specific multiple linear regression analyses were performed. For distal femoral hemiepiphysiodesis, change in mechanical lateral distal femoral angle (ΔmLDFA) was used as the dependent variable, whereas for proximal tibial hemiepiphysiodesis, change in mechanical proximal tibial angle (ΔmMPTA) served as the dependent variable. Age at surgery and treatment duration were included as independent variables in each model. Regression coefficients are reported with 95% confidence intervals. Model assumptions (linearity, homoscedasticity, absence of multicollinearity, and normal distribution of residuals) were verified.

To assess whether correction velocity differed by physis location, an additional multiple linear regression analysis was performed using annual correction rate (°/year) as the dependent variable. Physis location (distal femur vs. proximal tibia), age at surgery, and initial deformity magnitude were included as independent variables.

## 3. Results

Between 2012 and 2023, 99 limbs (68 patients, 130 physes) underwent guided growth surgery using a tension band plate for coronal plane angular deformity of the knee (distal femur and proximal tibia) and leg length discrepancy at our center. Data on 66 limbs (80 physes) was included, 22 (33 percent) of them with a neurological or osteological main disease. All the patients with a temporary epiphyseodesis due to leg-length-discrepancy were excluded (33 limbs). Eligibility required either skeletal maturity, documented by a closed growth plate at the left distal radius, or prior removal of the implant. Patients included in our study had a different range of aetiologies but all had coronal plane angular deformity of the knee as measured by mechanical axis deviation on the anteriorposterior radiograph. In total, 75.7 percent were male and the mean age was 11.9 (SD ± 2.2, range 6–15). The mean implant time was 1.75 years (SD ± 1.15 years, range 0.63–7.35). The mean follow up time was 6.4 years (SD ± 3.6, range 1.8–13.4).

We analyzed a total of 51 cases presenting with valgus deformity of the lower limb axis and 15 cases with varus deformity. All varus deformities were associated with an underlying neurological or osteological condition. In patients with valgus deformity, temporary hemiepiphysiodesis was performed at the distal medial femoral condyle in 72.5% of cases, at the proximal medial tibial plateau in 13.7%, and as a combined procedure in 13.7%. In patients with varus deformity, temporary hemiepiphysiodesis was performed at the proximal lateral tibial plateau in 40% of cases, at the distal lateral femoral condyle in 20%, and as a combined procedure in 40% (Table 2).

### 3.1. Underlying Conditions

Among the 22 procedures (limbs) with an underlying disorder, three had a neurological condition (two with Rett syndrome, one with spastic diplegia). The remaining patients suffered from osteological or metabolic disorders: five with achondroplasia, seven with X-linked hypophosphatemia, four with Morbus Fairbank (multiple epiphyseal dysplasia), and two with an unclassified metabolic disorder. In addition, one patient developed a post-traumatic varus deformity (Table 3).

### 3.2. Statistic Results

Therefore, two subgroups were defined for statistical analysis, as the outcomes of patients without underlying conditions could not be directly compared with those of patients presenting with the aforementioned disorders.

#### 3.2.1. Subgroup 1—Idiopathic (n 49 Physes)

The mechanical lateral distal femoral angle (mLDFA) increased from 82.8° preoperatively to 87.9° at treatment completion, yielding a mean change of +5.19° (SD ± 4.23; paired *t*-test t = 8.15, *p* < 0.001; Wilcoxon *p* < 0.001; paired Cohen’s d = 1.23). The mechanical proximal tibial angle (mMPTA) decreased from 90.5° to 88.6° (mean change −1.88°, SD ± 2.48; paired *t*-test t = −5.04, *p* < 0.001; Wilcoxon *p* < 0.001; Cohen’s d = −0.76). Corresponding correction rates were 4.75°/year for mLDFA and −1.74°/year for mMPTA.

In the idiopathic subgroup, 49 physes were derived from 24 unique patients (median 2 physes per patient, range 1–4). To account for within-patient clustering, a linear mixed-effects model was applied to the pre–post change in the relevant mechanical angle (femur: mLDFA; tibia: mMPTA). The model demonstrated a statistically significant mean angle change of +3.53° (95% CI 1.56 to 5.50; *p* < 0.001). The magnitude and direction of this effect were comparable to the results of the primary paired analysis that did not account for clustering, supporting the robustness of the findings. Collectively, these data indicate a clinically and statistically significant correction toward neutral mechanical alignment following temporary hemiepiphysiodesis in valgus deformity (Table 4).

In the distal femoral hemiepiphysiodesis group (n = 32 physes), multiple linear regression analysis demonstrated no statistically significant association between angular correction and either age at surgery (β = −0.96° per year; 95% CI −2.23 to 0.30; *p* = 0.13) or treatment duration (β = 0.003° per unit time; 95% CI −0.002 to 0.008; *p* = 0.22).

In the proximal tibial hemiepiphysiodesis group (n = 13 physes), neither age at surgery (β = 0.16° per year; 95% CI −1.70 to 2.02; *p* = 0.85) nor treatment duration (β = −0.008° per unit time; 95% CI −0.029 to 0.013; *p* = 0.42) was significantly associated with the magnitude of angular correction.

To further explore predictors of correction velocity, an additional multiple linear regression analysis was performed using the annual correction rate (°/year) as the dependent variable (n = 45 physes). Physis location (distal femur vs. proximal tibia), age at surgery, and initial deformity magnitude were included as independent variables. Physis location was not significantly associated with correction rate (β = −0.0042°/year for tibia vs. femur; 95% CI −0.0100 to 0.0016; *p* = 0.15). Similarly, neither age at surgery (β = −0.0006°/year; 95% CI −0.0028 to 0.0015; *p* = 0.57) nor initial deformity magnitude (β = 0.0007°/year; 95% CI −0.0003 to 0.0016; *p* = 0.18) showed a significant association with correction velocity.

#### 3.2.2. Subgroup 2—Neurological or Osteological Disorders, Posttraumatic

In the subgroup of patients with neurological or osteological comorbidities, statistical analysis was limited to descriptive evaluation due to the heterogeneous and difficult-to-interpret outcomes. Overall, only nine cases achieved the intended correction with primary treatment. In two patients with phosphate diabetes, repeated revisions and adjustments of the temporary hemiepiphysiodesis were required, as both overcorrection and additional procedures involving the tibia or femur became necessary. In the case of a young patient with Rett syndrome, the desired correction could not be achieved even at skeletal maturity. Similarly, correction could not be attained in a patient with post-traumatic varus deformity of the lower limb axis; this case, as well as that of a patient with an as-yet undiagnosed metabolic disorder, ultimately required corrective osteotomy. In the patient with valgus deformity and known Morbus Fairbank, the initial hemiepiphysiodesis at the proximal medial tibia and distal medial femur bilaterally resulted in overcorrection and had to be explanted and re-implanted in the opposite configuration (Table 5).

### 3.3. Complications

No major complications such as peri-implant infections or implant dislocations were observed in our cohort subgroup 1. We encountered only two cases of delayed wound healing and three cases in which postoperative pain was noticeably higher compared to the remaining patients. In these cases, the pain led to temporary limitations in mobility, which improved rapidly with physiotherapy. The described findings should not be considered complications. In particular, no peri-implant infections, implant breakage, or implant dislocation were observed in our cohort. The particular situations described above in patients with pre-existing osteological or neurological disorders were not classified as complications in this context.

## 4. Discussion

The present study demonstrates that guided growth using a tension band plate is an effective and safe technique for correction of coronal plane angular deformities around the knee in skeletally immature patients. Descreptive datas are summarized at Table 6. Significant correction of both distal femoral valgus and proximal tibial valgus deformities was achieved, with mean changes of +5.19° in the mLDFA and −1.88° in the mMPTA, corresponding to correction rates of 4.75°/year and −1.74°/year, respectively. These findings are consistent with previous reports describing annual correction rates between 4° and 6° for the distal femur and 2° to 4° for the proximal tibia following temporary hemiepiphysiodesis using tension band plates [4,6,7,15].

The magnitude and rate of correction in our idiopathic subgroup align closely with those reported by Stevens et al. [4]. and Burghardt et al. [6], who observed mean correction rates of 0.4–0.6°/month in distal femoral valgus deformities. Similarly, Ballal et al. [7]. reported an average correction rate of 0.5°/month using the Eight-Plate^®^ system, emphasizing the predictable and progressive nature of this technique. Our results confirm these findings and support the use of guided growth as a standard of care for idiopathic coronal plane deformities in growing children.

Results of subgroup 1 with idiopathic coronal plane deformities suggest that, within this cohort, the achieved correction of the mechanical axis after temporary hemiepiphysiodesis was not significantly influenced by patient chronological age or treatment duration. Other factors, such as growth potential or individual variations in physeal response, may contribute stronger to the overall correction.

The subgroup with underlying neurological or osteological disorders demonstrated less consistent outcomes, which is in line with previous studies suggesting slower and less predictable correction in pathologic physes [16,17,18]. Our results of subgroup 2 underscore that in patients with complex neurological or osteological comorbidities, temporary hemiepiphysiodesis may have limited effectiveness and often requires individualized treatment strategies or alternative surgical approaches. The reduced responsiveness in this cohort may be attributed to altered physeal biology or asymmetric growth potential resulting from the underlying condition. These results reinforce the importance of differentiating idiopathic from pathological deformities when assessing treatment outcomes.

Several factors appear to influence the correction rate and final outcome. In line with prior studies [19,20], younger patients tended to achieve faster correction, likely due to higher residual growth potential. Furthermore, deformities treated at the distal femur corrected more rapidly than those treated at the proximal tibia, consistent with the greater contribution of the distal femoral physis to overall limb growth. These observations underline the importance of careful timing and site selection in guided growth procedures to optimize correction before skeletal maturity. Consistent with the findings of Morasiewicz et al., our cohort demonstrated that patient sex had no significant influence on the radiographic correction or overall clinical outcome. This suggests that the biological mechanisms underlying guided growth are largely independent of sex-specific differences and that treatment efficacy can be expected to be comparable in male and female patients [21].

The recent literature continues to emphasize the critical importance of timing in the removal of tension-band plates to balance correction and rebound risk. Xiao et al. recommend delaying plate removal until a modest overcorrection (around 5°) is achieved, based on growth-prediction models, to mitigate rebound [22]. Complementing this, a 2023 study by Aksoy et al. demonstrated that overcorrected joint-orientation angles frequently regress toward neutrality over time, with most segments returning to physiological alignment and only a minority requiring revision [23]. Moreover, the “sleeper-plate” technique, in which only the metaphyseal screw is removed, has been shown to preserve achieved correction in about half of patients over a mean follow-up of 3.5 years; however, rebound and tethering remain a concern, particularly in younger children [24]. Given these findings, a “watchful waiting” strategy—maintaining close radiographic surveillance until neutral or slightly overcorrected alignment and minimal residual growth—is increasingly supported to minimize both over- and under-correction.

No major complications such as hardware failure, infection, or permanent growth plate damage were observed. Minor implant-related irritations and screw loosening occurred infrequently, consistent with rates reported in the literature [6]. Following implant removal, mild rebound of deformity was observed in some patients, especially those treated at a younger age or with underlying neurological conditions. This phenomenon has been described by several authors [25,26] and highlights the necessity of continued follow-up after plate removal to prevent over- or undercorrection.

The findings of this study confirm that guided growth with a tension band plate provides a reliable, minimally invasive, and reversible technique for gradual correction of coronal plane deformities. It allows for precise modulation of growth while avoiding the need for osteotomy, thereby minimizing morbidity and recovery time. Nevertheless, individualized treatment planning remains essential, particularly in patients with systemic or neurological conditions, where correction may be slower and incomplete.

### Limitations

This study has several limitations. First, its retrospective design is inherently associated with potential selection bias, as treatment decisions and follow-up assessments were based on clinical practice rather than predefined study protocols.

Second, the patient population was heterogeneous, particularly within the pathological subgroup, which comprised a wide range of neurological, osteological, and metabolic conditions with differing underlying pathophysiology and growth behavior. This heterogeneity limits the comparability of outcomes within this subgroup and restricts the interpretability of subgroup-specific effects. Functional outcomes such as gait improvement or patient-reported outcome measures were not systematically collected, which precludes conclusions regarding functional or quality-of-life–related benefits beyond radiographic correction.

A further limitation of this study is the small sample size of the pathological subgroup (*n* = 15), which consisted exclusively of patients with underlying conditions and therefore limits the generalizability of the varus correction results to patients with idiopathic deformities.

Future prospective studies should integrate standardized functional assessments, patient-reported outcomes, and radiographic analyses in larger and more homogeneous cohorts to better quantify the long-term benefit of guided growth procedures.

## 5. Conclusions

In conclusion, temporary hemiepiphysiodesis using a tension band plate is a highly effective and predictable technique for the correction of idiopathic coronal plane deformities of the knee in growing children. The correction rates observed in this study are comparable to previously published data. Although outcomes in patients with neurological or osteological comorbidities were less favorable, guided growth remains a valuable option for gradual correction in this challenging population. Continuous and tight postoperative monitoring are essential to detect rebound phenomena and ensure optimal long-term alignment.

## Figures and Tables

**Table 1:** Definitions of angular measurements used for deformity analysis.

Abbreviation	Full Term	Definition	Normal Range (°)
mLDFA	Mechanical Lateral Distal Femoral Angle	Lateral angle between the mechanical axis of the femur and the distal femoral joint line.	88° (85°–90°)
mMPTA	mechanical Medial Proximal Tibial Angle	Medial angle between the mechanical axis of the tibia and the proximal tibial joint line.	87° (85°–90°)

**Table 2:** Distribution of surgical indications for coronal plane deformities of the lower limb.

Deformity Type	Total Cases (n)	Surgical Intervention	Distribution (%)
Valgus deformity	51	Distal medial femoral hemiepiphysiodesis	72.5%
		Proximal medial tibial hemiepiphysiodesis	13.7%
		Combined hemiepiphysiodesis	13.7%
Varus deformity	15	Proximal lateral tibial hemiepiphysiodesis	40.0%
		Distal lateral femoral hemiepiphysiodesis	20.0%
		Combined hemiepiphysiodesis	40.0%

**Table 3:** Details of underlying disorders.

Category	Specific Diagnosis	Limbs (n)	Percentage (%)
Neurological disorders	Rett syndrome	2	9.1%
Neurologic disorders	Spastic diplegia	1	4.5%
Osteological disorders	Achondroplasia	5	22.7%
	Morbus Fairbank (multiple epiphyseal dysplasia)	4	18.2%
Metabolic disorders	X-linked hypophosphatemia	7	31.8%
	Unclassified metabolic disorder	2	9.1%
Post-traumatic	Varus deformity	1	4.5%
Total		22	100%

**Table 4:** Significant results for mLDFA and mMPTA. Significant results highlighted bold.

Parameter	Pre-op Mean (SD)	Post-op Mean (SD)	Mean Change (SD)	Paired *t*-Test (*p*)	Wilcoxon (*p*)	Cohen’s d	Correction Rate (°/Year)
mLDFA (°)	82.75	87.94	+5.19 (4.23)	t = 8.15, *p* < 0.001	***p* < 0.001**	1.23	4.75 (3.69)
mMPTA (°)	90.51	88.62	−1.88 (2.48)	t = −5.04, *p* < 0.001	***p* < 0.001**	−0.76	−1.74 (2.46)

**Table 5:** Overview of unexpected failure in subgroup 2 with underlying conditions.

Underlying Condition	No. of Limbs	Primary Correction Achieved	Additional Procedures/Revisions	Final Outcome
Phosphate diabetes	2	No	Repeated revisions; alternating overcorrection; additional femoral and tibial procedures	Partial correction
Rett syndrome	1	No	None	No correction achieved at skeletal maturity
Post-traumatic deformity	1	No	Corrective osteotomy	Osteotomy required
Suspected metabolic disorder	1	No	Corrective osteotomy	Osteotomy required
Morbus Fairbank	1	No	Bilateral explantation and re-implantation in opposite configuration due to overcorrection	Correction after revision

**Table 6:** Patient characteristics and surgical details—Summary.

Parameter	Valgus Deformity (*n* = 51)	Varus Deformity (*n* = 15)
Mean age (years, SD, range)	11.8 (SD ± 2.2, 6–15)	12.1 (SD ± 2.3, 7–15)
Male (%)	76%	80%
Underlying neurological/osteological disease (%)	27.5%	100%
Implant location	Distal medial femur: 72.5%Proximal medial tibia: 13.7%Combined: 13.7%	Proximal lateral tibia: 40%Distal lateral femur: 20%Combined: 40%
Mean implant duration (years, SD, range)	1.75 (SD ± 1.15, 0.63–7.35)	1.75 (SD ± 1.15, 0.63–7.35)
Mean follow-up (years, SD, range)	6.4 years (SD ± 3.6, 1.8–13.4)	6.4 (SD± 3.6, 1.8–13.4)

## Data Availability

The collected data are properly stored in the study archive of the Department of Orthopaedics and Traumatology at Kepler University Hospital and protected from misuse by third parties.

## Abbreviations

The following abbreviations are used in this manuscript:

mLDFA	Mechanical Lateral Distal Femoral Angle
mMPTA	Mechanical Medial Proximal Tibial Angle
SD	Standard Deviation
IQR	Interquartile range
CI	Confidence interval
